# Sustained release Curcumin loaded Solid Lipid Nanoparticles

**DOI:** 10.15171/apb.2016.04

**Published:** 2016-03-17

**Authors:** Parisa Jourghanian, Solmaz Ghaffari, Mehdi Ardjmand, Setareh Haghighat, Mahdieh Mohammadnejad

**Affiliations:** ^1^ Department of Chemical Engineering, Shahrood Branch, Islamic Azad University, Shahrood, Iran.; ^2^ Department of Medical Nanotechnology, Faculty of Advanced Sciences and Technology, Pharmaceutical Sciences Branch, Islamic Azad University, (IAUPS), Tehran, Iran.; ^3^ Young Researchers and Elite Club, Pharmaceutical Sciences Branch, Islamic Azad University, (IAUPS), Tehran, Iran.; ^4^ Department of Chemical Engineering, South Tehran Branch, Islamic Azad University, Tehran, Iran.; ^5^ Department of Microbiology, Faculty of Advanced Sciences and Technology, Pharmaceutical Sciences Branch, Islamic Azad University (IAUPS), Tehran, Iran.; ^6^ Quality Control Department, Tofigh Daru Research and Engineering Co. Tehran, Iran.

**Keywords:** Curcumin, Solid Lipid Nanoparticles, Sustained release, Freeze drying, Particle size

## Abstract

***Purpose:*** curcumin is poorly water soluble drug with low bioavailability. Use of lipid systems in lipophilic substances increases solubility and bioavailability of poorly soluble drugs. The aim of this study was to prepare curcumin loaded Solid Lipid Nanoparticles (SLNs) with high loading efficiency, small particle size and prolonged release profile with enhanced antibacterial efficacy.

***Methods:*** to synthesize stable SLNs, freeze- Drying was done using mannitol as cryoprotectant. Cholesterol was used as carrier because of good tolerability and biocompatibility. SLNs were prepared using high pressure homogenization method.

***Results:*** optimized SLNs had 112 and 163 nm particle size before and after freeze drying, respectively. The prepared SLNs had 71% loading efficiency. 90% of loaded curcumin was released after 48 hours. Morphologic study for formulation was done by taking SEM pictures of curcumin SLNs. Results show the spherical shape of curcumin SLNs. DSC studies were performed to determine prolonged release mechanism. Antimicrobial studies were done to compare the antimicrobial efficacy of curcumin SLNs with free curcumin. DSC studies showed probability of formation of hydrogen bonds between cholesterol and curcumin which resulted in prolonged release of curcumin. Lipid structure of cholesterol could cause enhanced permeability in studied bacteria to increase antibacterial characteristics of curcumin.

***Conclusion:*** the designed curcumin SLNs could be candidate for formulation of different dosage forms or cosmeceutical products.

## Introduction

Recently phytotherapeutics were prepared in nano size to improve pharmacokinetic and pharmacodynamics characteristics.^1^ Curcumin, a phenolic compound from the plant Curcuma Longa as a traditional spice have many pharmacological activities including: anti-diabetic, anti-inflammatory, anti-cancer, anti-oxidant, antibacterial, anti-HIV and anti-aging activity and hepato protective activity as well as cardiovascular benefits.^2^ According to Jadhav et al (2014), curcumin as a multifunctional phytoceutical, has been patented numerously.^1^ Studies demonstrated that curcumin acts on multiplemolecular targets to selectively kill tumour cells with low intrinsic toxicity.^1,3-5^ This compound is widely used in China, India and Iran as a spice and traditional herb.^6^ The unexpected discovery that curcumin is also a powerful iron chelator has given new insight into its multimodal mechanisms of action in gaining control of age-related iron accumulations in the brain, heart, and liver.^7^ Curcumin is a poorly water-soluble drug with low oral bioavailability and sensitive to photodegradation. Application of lipids for bioavailability enhancement of poor soluble drugs is promising. Since most of the components involved in formulation of solid lipid nanoparticles are of natural origin, they are compatible with components of biological membrane, and generally less toxicological risk is experienced.^8^

Nanoparticles (NPs) are colloidal particles in which the active ingredients are dissolved, entrapped in and/or is adsorbed or attached on to the particle.^9^ Some advantages of nanotechnology in phytoceuticals include: the probability of designing sustained release systems, enhanced physicochemical stability, enhanced permeability, improves tissue distribution and enhanced solubility and bioavailability.^1^ Many novel drug delivery strategies such as liposomes, nano- or micro- emulsions, polymeric NPs and solid lipid NPs, polymer conjugates, polymeric micelles, nanocrystals, nanogels, self-assemblies and cyclodextrin inclusion complexes have been described to increase solubility, bioavailability and delivery of curcumin.^10^

Solid lipid nanoparticles (SLNs) as colloidal carrier systems combine the advantages of traditional systems but avoid some of their major disadvantages. These colloidal systems have many important advantages, such as biocompatibility, good tolerability, and ease of scale-up.^11,12^ SLNs have been studied as a drug-delivery system for the controlling of drug release.^13^ Although SLNs used as carrier to sustain the release of lipophilic drugs but there are studies using SLN(s) for the delivery of hydrophilic drugs and peptides too. SLNs have been reported as drug carriers to treat neoplasms and tumour targeting.^9^ Many studies were done to increase stability, solubility and efficacy of curcumin using nanotechnology.^9,14^ Some studies were carried out to evaluate benefit of SLNs as a carrier system for curcumin for treatment of arthritis.^15^ The findings emphasize that SLNs could be novel approach to deliver curcumin into the inflamed joints and improve its biopharmaceutical performance.^15^ Curcumin SLNs were designed for treatment of lung cancer by Wang et al 2013.^16^ As curcumin has been used widely in Iran, in the present study, curcumin loaded SLNs were synthesized to prolong drug release and enhance antibacterial effects.

## Materials and Methods

### Materials

Tween 80, ethanol and acetone were purchased from Merck, Germany and were used as surfactant and oily phase solvents, respectively. Curcumin was prepared by Sami Lab, India. Dialysis membrane was purchased from Sigma, Germany. All ingredients which were used to prepare phosphate buffer solution were purchased from Merck, Germany.

### Preparation of Curcumin SLNs

Curcumin loaded SLNs were prepared using high pressure homogenization. 0.1 g tween 80 and 600 mg curcumin were added to 10 ml of purified water at room temperature, 600 mg of cholesterol was dissolved in mixture of ethanol and acetone on 3-1 ratio at 75-80 °C, then the hot oily phase was added to aqueous phase under homogenization at 11000 rpm and the mixture was homogenized for 7 minutes. While the mixture cooled to room temperature, SLNs formed.^13^

### Particle size analysis

The evaluation of particle size was done using Malvern zeta sizer (ZEN3600) and confirmed by scanning electron microscopy (SEM).

### Measurement of drug loading efficiency

For determination of drug loading efficacy (LE%) of curcumin solid lipid nanoparticles, the samples were centrifuged at 26,000 rpm for 35 minutes at -4 °C using Sigma laboratories centrifuge (Germany). The drug concentration in the supernatant was analyzed using UV spectrophotometer (Shimadzu, Japan) at 424 nm and LE% was calculated using reverse method applying following equation.^17^

$$Drug\;Loading\;efficacy\;\left(LE\%\right)=\frac{Drug_{total}-Drug_{\text{sup}erna\text{tan}t}}{Drug_{total}}\times 100$$

### Drug release study

Release study was performed using dialysis sack method by DO405 dialysis tubing 23-15mm (Sigma, Germany). 5 ml of each formulation was placed in dialysis membrane and immersed in 50 ml phosphate buffer pH 7.4 containing 0.1% Tween 80. Two ml samples were withdrawn in predetermined time intervals and drug concentration was measured at 424 nm using UV spectrophotometer. To ensure that sustained release profile is not due to membrane, curcumin dispersion in the same concentration with curcumin SLNs was studied under the same condition for release.

### Morphology Study

Morphology of the nanoparticles was characterized by scanning electron microscopy (SEM). The nanoparticles were mounted on aluminum stubs; sputter- coated with a thin layer of Au-Pd and examined using an SEM (SEM XL30, Philips, Netherlands).

### Freeze drying

To increase stability and shelf life, curcumin SLNs were lyophilized successfully. Lyophilization was done by using 5 and 15% mannitol as cryoprotectan at freezing temperature equal to -20ᴼ C for 24 hours and then particles were lyophilized for 48 hours by freeze-dryer (Lyotrap/ Plus,UK) at -40ᴼ C and pressure equal to 0.4 bar.

### DSC Analysis

DSC thermograms were obtained using (Mettler, Switzerland). Certain amount of dried nanoparticle powder was crimped in a standard aluminum pan and heated from 25 to 400 °C at a heating rate of 10 °C/min under constant heating.

### Antimicrobial Efficiency

To compare the antimicrobial activity of curcumin loaded nanoparticles with free curcumin, ‘‘well diffusion test’’ was carried out using E. coli (ATCC 25922) as the Gram-negative pathogenic strain and S. aureus (ATCC 25923) as Gram positive strain. In this study SLNs without curcumin was studied as blank. The antibacterial effect of curcumin against many bacterial strains including E.coli, S. aureus, S. epidermidis was reported by Zorofchian et al 2014. In this study the antibacterial efficiency of curcumin loaded SLNs was compared with the same concentration of free curcumin.^18^

## Results and Discussion

### Particle size

**Figure 1:**
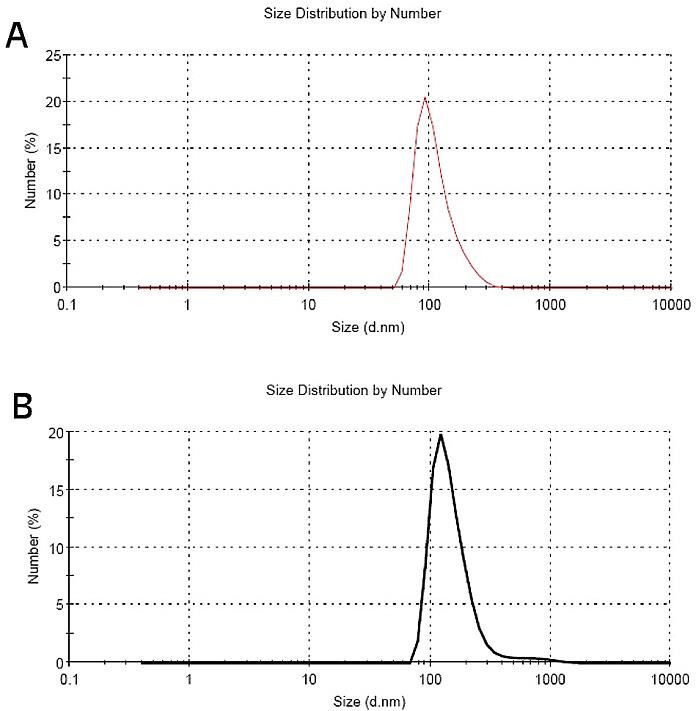
Particle size distribution of curcumin solid lipid nanoparticles before (A) and after (B) freeze drying.

Particle size studies demonstrated that particles were 112 nm with PdI (Polydispersity index) equal to 0.114 before freeze drying. The particle size of nanoparticles was 163 and 306 nm when 5% and 15% mannitol was used as cryoprotectant, respectively. Therefore 5% mannitol was chosen as optimum cryoprotectant for further experiments. Particle size studies establish good size distribution before and after freeze drying. Figure 1a and 1b show the result of particle size distribution before and after freeze drying, respectively.

### Morphology study

**Figure 2:**
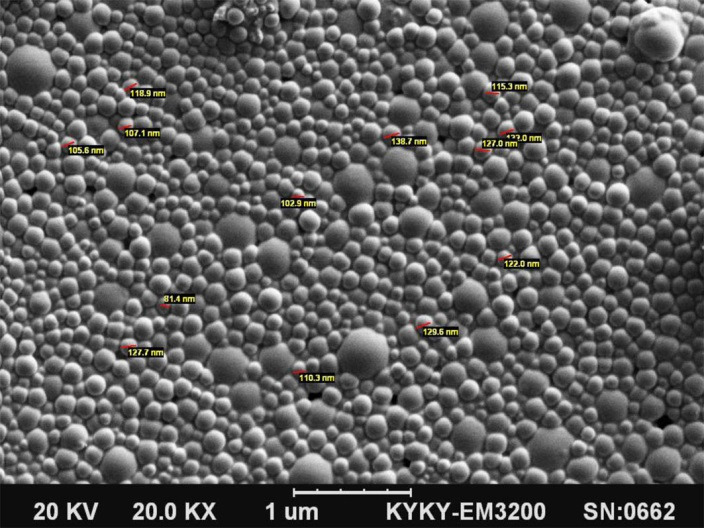
Scanning electron microscopy picture of curcumin solid lipid nanoparticles.

SEM photograph of curcumin loaded SLNs was shown in Figure 2. According to SEM image, prepared SLNs showed spherical shape. The size distribution of particles was comparable to DLS.

### Loading efficiency and drug release study

**Figure 3:**
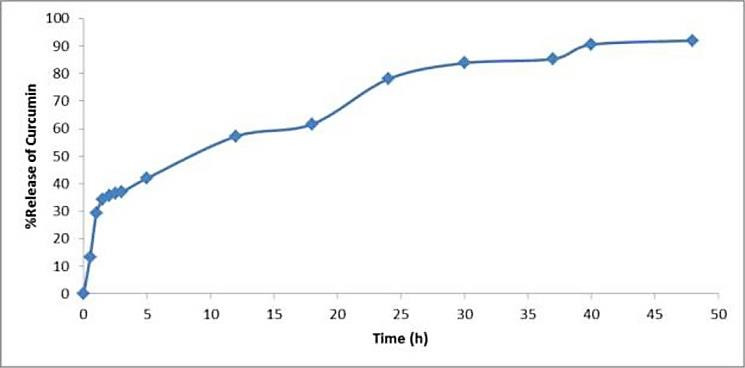
Curcumin release profile through SLNs.

Using equation 1, loading efficiency was calculated as 70.4±1.2%. Figure 3, shows curcumin release profile. Release profile of curcumin from nanoparticles shows a prolonged manner. Results show that after 36 and 48 hours more than 85% and 92% of loaded curcumin released, respectively. More than 95% of curcumin released from free curcumin dispersion after less than one hour through dialysis membrane. Chen et al prepared prolonged release curcumin using liposomes as carrier, in their study after 48 hours less than 70% of loaded curcumin was released.^19^ Other study was done to prepare unilamellar liposomes of curcumin analogue to increase hepatoprotective efficacy, in this study the active ingredients release was prolonged for 10 days.^20^

### DSC studies

**Figure 4:**
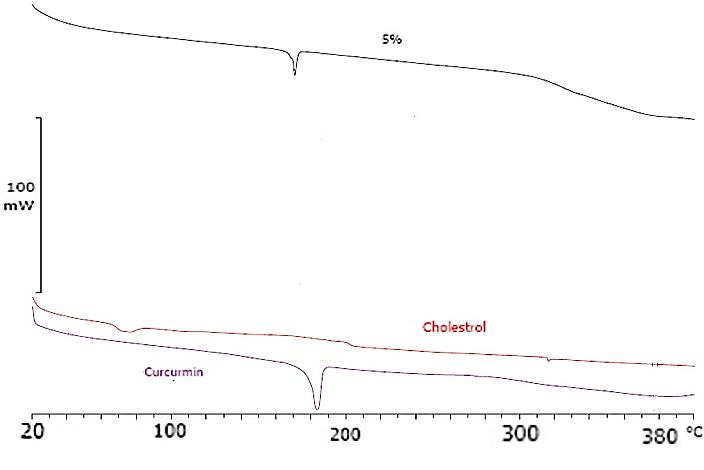
DSC thermograms of raw materials and freeze-dried curcumin SLNs.

Figure 4 shows DSC analysis results. DSC thermograms of pure cholesterol, curcumin and curcumin-loaded SLNs show that melting point of cholesterol has shifted from 80 °C to about 170 °C in the SLNs of curcumin. The melting temperature of curcumin is also seen at 185 °C in the thermogram of pure curcumin. Chemical structure of curcumin and cholesterol could support probability of hydrogen band formation between these two compounds which resulted in prolonged release from nanoparticles.

### Antimicrobial effect study

**Figure 5:**
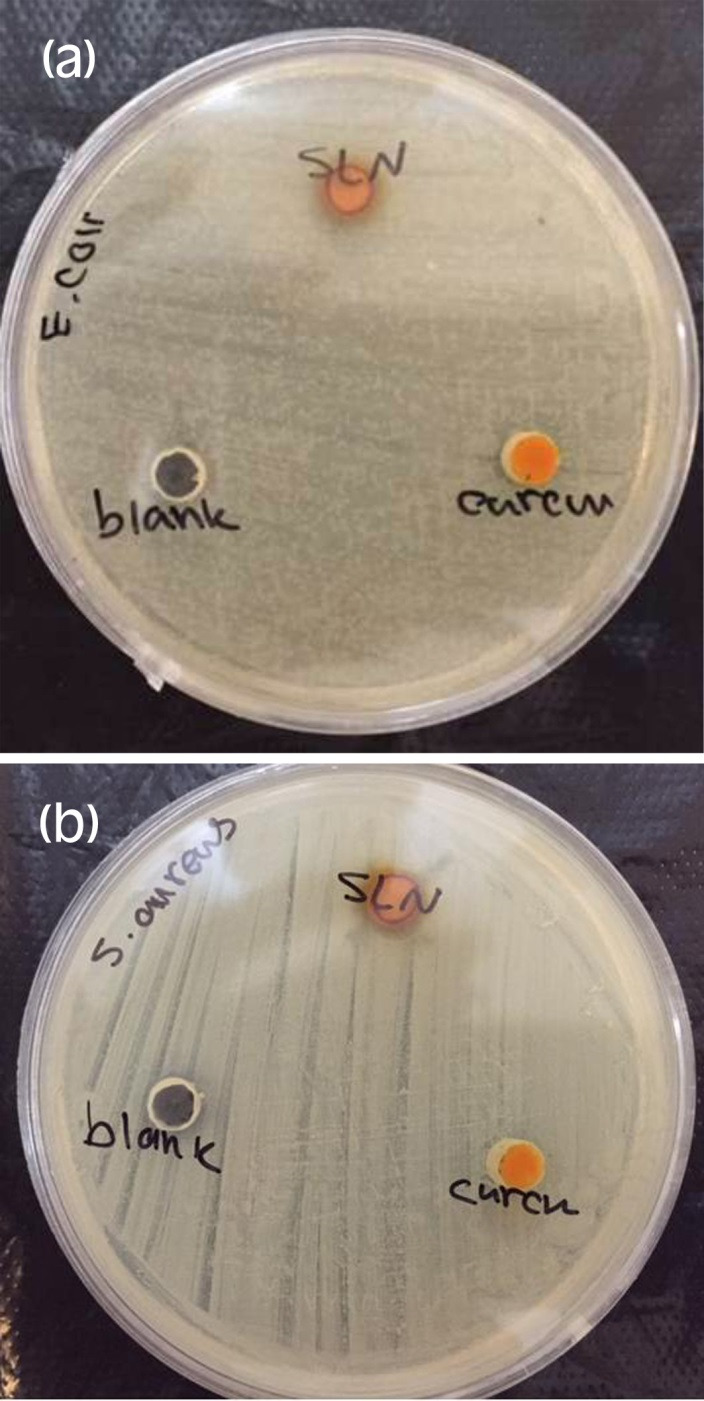
Photographs of the zone of inhibition produced by free curcumin and its SLNs against E.coli (a) and S. aureus (b).

Figures 5a and 5b show increased antimicrobial effect of curcumin nanoparticles compared to free curcumin in same concentration on E.coli and S. aureus. In another study, the synergistic effect of curcumin and other antibacterial agents was observed.^21^ Based on our findings, curcumin SLNs can be combined with other antibiotics in SLNs to reduce the MIC (Minimum Inhibitory Concentration) and MBC (Minimum Bactericidal Concentration) more. The reduction of MIC and MBC of some antibacterial agents loaded in SLNs was demonstrated before.^12,22^

## Conclusion

Our results show that curcumin SLNs can be prepared and freeze dried with proper size, drug loading and release profile. Curcumin SLNs can be used in sustained release oral dosage forms to increase bioavailability, topical formulations for wound dressing, or anti-aging products. Since the particles are in proper size range, it is predictable that particles show enhanced permeability at site of action. Also the designed curcumin SLNs could be used to reach more MIC and MBC reduction in combination with other antibiotics for synergistic effects. According to Ronita et al study (2009) curcumin is effective against H. pylori,^23^ so curcumin SLNs can be used with therapeutic protocols of H. pylori treatment.

## Acknowledgments

Authors would like to thank Shahrood Branch and Pharmaceutical Sciences Branch, Islamic Azad University for financial and technical support and Tofigh Daru Co for technical support of this project. Authors are also grateful to Miss Hedieh Ghaffari for English editing of the manuscript and Dr. Tarane Gazori for scientific editing of the manuscript.

## Ethical Issues

Not applicable.

## Conflict of Interest

Authors declare no conflict of interest in this study.
